# Revolutionizing core muscle analysis in female sexual dysfunction based on machine learning

**DOI:** 10.1038/s41598-024-54967-0

**Published:** 2024-02-27

**Authors:** Doaa A. Abdel Hady, Tarek Abd El-Hafeez

**Affiliations:** 1 Department of Physical Therapy for Women’s Health, Faculty of Physiotherapy, Deraya University, EL-Minia, Egypt; 2 Department of Computer Science, Faculty of Science, Minia University, EL-Minia, Egypt; 3 Computer Science Unit, Deraya University, EL-Minia, Egypt

**Keywords:** Core muscle analysis, Female sexual dysfunction, Machine learning, Deep learning, Rehabilitation, Health care, Health occupations, Computer science, Information technology, Scientific data

## Abstract

The purpose of this study is to investigate the role of core muscles in female sexual dysfunction (FSD) and develop comprehensive rehabilitation programs to address this issue. We aim to answer the following research questions: what are the roles of core muscles in FSD, and how can machine and deep learning models accurately predict changes in core muscles during FSD? FSD is a common condition that affects women of all ages, characterized by symptoms such as decreased libido, difficulty achieving orgasm, and pain during intercourse. We conducted a comprehensive analysis of changes in core muscles during FSD using machine and deep learning. We evaluated the performance of multiple models, including multi-layer perceptron (MLP), long short-term memory (LSTM), convolutional neural network (CNN), recurrent neural network (RNN), ElasticNetCV, random forest regressor, SVR, and Bagging regressor. The models were evaluated based on mean squared error (MSE), mean absolute error (MAE), and R-squared (R^2^) score. Our results show that CNN and random forest regressor are the most accurate models for predicting changes in core muscles during FSD. CNN achieved the lowest MSE (0.002) and the highest R^2^ score (0.988), while random forest regressor also performed well with an MSE of 0.0021 and an R^2^ score of 0.9905. Our study demonstrates that machine and deep learning models can accurately predict changes in core muscles during FSD. The neglected core muscles play a significant role in FSD, highlighting the need for comprehensive rehabilitation programs that address these muscles. By developing these programs, we can improve the quality of life for women with FSD and help them achieve optimal sexual health.

## Introduction

Female sexual dysfunction (FSD) encompasses a spectrum of sexual health challenges that can influence women across various age groups^1^. This condition holds the potential to profoundly affect a woman's overall quality of life, interpersonal relationships, and self-esteem. FSD may manifest in diverse ways, such as a diminished desire for sexual activity, difficulties in attaining or sustaining sexual arousal, challenges reaching orgasm, discomfort or pain during intercourse, and other associated symptoms^2^. FSD is not a rare condition, with prevalence ranging from 38 to 85.2%^3,4^.

The multifaceted nature of FSD implies that it is not solely a physical or psychological concern but often a complex interplay of both factors. Physical causes contributing to FSD may involve hormonal imbalances, side effects of medications, chronic illnesses, and alterations in the body attributed to aging or menopause. Concurrently, psychological factors, including stress, anxiety, depression, relational issues, and experiences of trauma, can significantly contribute to the onset or exacerbation of FSD. Understanding FSD as a holistic issue necessitates recognizing the myriad factors that can influence its development. By acknowledging both the physical and psychological aspects, healthcare professionals can adopt a comprehensive approach to diagnosis and treatment, addressing the diverse factors that may contribute to FSD in women^5^.

It is well known that the prevalence of sexual dysfunction in female due to pelvic floor muscle (PFM) dysfunction, suggesting a possible anatomical correlation between PFM function and female sexual function^6^, as the PFM react to sexual stimuli with increased local blood circulation and involuntary contractions during orgasm. So, the weakness of pelvic muscles could contribute to the inability of a woman to achieve orgasm. Training the pelvic floor muscles helps improve female sexual function, in all its domains Their contraction has been linked to increased sexual and orgasmic responses^7^.

Traditionally, the assessment of core muscle function during sexual activity has been limited to subjective self-report measures or invasive procedures, such as electromyography. However, recent advances in machine and deep learning have opened up new possibilities for non-invasive and objective assessment of core muscle function^8^.

There is a lack of knowledge on core muscles and PFM dysfunction, especially in FSD. It would be very crucial for predicting core muscle changes in women during sexual dysfunction to enhance decision-making during physical therapy for pelvic floor rehabilitation. The findings of this study have important implications for understanding the relationship between core muscles and FSD and may help develop targeted interventions to improve sexual function in women with FSD. This study also highlights the potential of the machine and deep learning models in predicting changes in core muscles, which may have broader applications in other areas of healthcare.

### Problem statement

The ability to accurately predict changes in core muscle activity during FSD using non-invasive medical imaging could help clinicians better diagnose and manage patient conditions. However, developing predictive models for such a task poses several challenges, including processing diverse imaging data, extracting meaningful features, and accounting for variability across individuals.

While prior studies have applied machine and deep learning regression techniques to assess core muscles, comparisons of different algorithms on this specific prediction problem are still limited. Additionally, feature selection approaches to optimize models for clinical usage have not been systematically evaluated.

Therefore, this study aims to address the problem of predicting changes in pelvic floor and associated core muscles during FSD by:

1. Comparing the performance of various regression machine and deep learning algorithms
2. Evaluating different feature selection techniques to identify the most predictive inputs
3. Assessing model accuracy, interpretability and computational efficiency

The goal is to determine the most suitable algorithm(s) and features for developing an accurate, clinically-viable predictive solution to aid diagnosis and monitoring of FSD patients based on non-invasive imaging analysis. This could help advance effective, personalized treatment strategies for improving women's health outcomes.

### Contributions

In this study, we constructed and deployed five machine learning regression models—comprising ElasticNetCV, random forest regressor, Support Vector Regressor (SVR), Bagging regressor, and decision tree regressor—to estimate the transverse abdominis (TrA) ratio, maximal foreward stroke (MF) ratio, pelvic floor muscle (PFM) force, and diaphragm excursion. To compare the performance of these models, we utilized mean squared error (MSE), mean absolute error (MAE), and R-squared Score as evaluation metrics. We investigated the fitting time needed for each model to gauge their computational complexity and scalability. Results were subsequently organized in tables and graphs for clarity, enabling us to distinguish the finest models based on prediction accuracy and computational demands.

Through correlation analyses, we detected robust positive links between MF Ratio and TrA ratio, as well as MF CONT and MF REST, revealing a close association among these features. Additional moderate positive correlations surfaced between the diaphragm attribute and VLQ, FSFI, TrA R, TrA c, PFM Force, TrA ratio, and MF Ratio. To streamline the regression modeling, we engaged feature selection techniques, such as F-value selector, mutual information selector, RFE with logistic regression, random forests-derived feature selection, and variance thresholding. Our analysis highlighted the consistent high rankings of 'Diaphragm', 'PFM force', 'FSFI', and 'VLQ' features across various methodologies.

## Materials and methods

### Trial design

This study was designed as an observational and cross-sectional study and was approved by the Ethical Committee at Deraya University, El-Minya, Egypt (No: 6/2023). The study adhered to the ethical standards outlined in the Declaration of Helsinki and complied with principles for human research. All patients provided written consent after receiving a thorough description of the trial. The study was conducted at an outpatient clinic between February 1, 2023, and April 15, 2023. The clinical trial identifier for this study is NCT/05833685.

### The sample size

The study initiated a sample size calculation before its commencement to mitigate the risk of type 2 errors. The estimated sample size was determined using the software tool G*Power^9^. Based on statistical indices, with an effect size (dz) of 0.5, an α error probability of 0.05, a power analysis (1-B) error of 0.95, and a two-sided 5% significance level, the total estimated sample size for the study was determined to be at least 45 women, with 50 women allocated to each group, accounting for potential dropouts. Actually, the effect size of 0.5 is considered medium in Cohen's d convention, and it is frequently adopted in social sciences research. Since our study addresses the improvement of pelvic floor muscle function, which shares similarities with psychotherapy and rehabilitation studies, we opted for the medium effect size as a realistic assumption. Moreover, previous literature on pelvic floor disorders has not established universally accepted guidelines for effect size, so we chose the most commonly used level in analogous fields.

### Eligibility criteria

Group A consists of 50 females who have been diagnosed with FSD based on the Female Sexual Function Index (FSFI) assessment, whilst Group B contains 50 healthy females meeting specific criteria, i.e., aged between 30 and 40 years, with a Body Mass Index (BMI) between 25 and 30 kg/m^2^, a maximum of three normal deliveries, and regular menstruation.

### Exclusion criteria

Women with a medical history of disc prolapse, sacroiliac joint issues, symphysis pubic joint problems, lower limb problems, urinary incontinence (UI), lower urinary tract symptoms, neurological diseases, diabetes mellitus, smoking habits, cognitive deficits, genital prolapse, leg length discrepancy, diastasis recti, diabetes, use of intrauterine devices, and previous surgeries related to the spine, abdomen, or pelvis were excluded from participation. Additionally, women using medications for pain or UI, as well as those taking drugs for sexual dysfunction or medications that affect collagen or healing (such as chemotherapy drugs, psychotropic medications, corticosteroids, and anti-inflammatory medications), were also not eligible for participation.

### Evaluation procedures

Evaluation of two groups (A, B).

#### Assessment of pelvic floor function

The evaluation of pelvic floor muscle (PFM) thickness and strength in all patients was performed using an ultrasound imaging unit (Mindray DP10, B-mode, Serial number: bn-75013216, China) equipped with a convex transducer operating at a frequency of 5 MHz. This imaging unit has demonstrated reliable results with good inter-rater reliability for measuring PFM thickness (ICC: 0.81) and PFM force (ICC: 0.7123), as well as good intra-rater reliability (ICC: 0.98 for PFM thickness and ICC: 0.9841 for PFM force) respectively^10^.

During the measurements, the participants were positioned in the crook position with their lumbar spine in a neutral position, and their hips and knees bent at a 60° angle. The ultrasound transducer was inserted transversely across the midline of the abdomen, directly above the symphysis pubis, at an approximate angle of 60° from the vertical^11^. To ensure an accurate examination plan, the participants were asked to relax their pelvic floor muscles (PFM) and then perform a maximum contraction. A marker (X) was placed on the image of the bladder at the junction of the hyper and hypoechoic structures. Another marker was placed at the end of the muscle, and the measurement was taken as the distance between these two points. It is important to note that these markers were used to facilitate accurate measurements^12^.

Following the initial practice session, the women performed three maximum pelvic floor muscle (PFM) contractions to measure the displacement of the posterior bladder wall caused by the PFM contraction. A clearly defined edge, consistently visible throughout the movement, was chosen for measurement at the point of the greatest observed displacement. The image was captured at the moment of maximum displacement, after which the woman relaxed her PFM. The investigator, who was blinded to the measurement value, then measured the displacement from its current position in the static image. The transducer was kept in a fixed position throughout the procedure to maintain a constant field of vision between rest and maximal contraction. The mean of the three measurements performed by the same investigator was used for subsequent statistical analysis^12^.

#### Assessment of diaphragmatic excursion

Using a supine position, a 2.5–5 MHz curvilinear transducer in M-mode ultrasound imaging was employed to assess Diaphragmatic excursion in all female subjects. Positioned between the mid-clavicular and anterior axillary lines, below the right costal margin, the probe was oriented medially, cephalically, and dorsally to capture the posterior aspect of the right hemi-diaphragm. Diaphragmatic excursion measurements involved placing calipers at the lower and upper points of the inspiratory slope, with all measurements recorded after the expiration phase^13^.

Diaphragmatic excursion ultrasound offers excellent temporal resolution, remarkable reproducibility, and exceptional accuracy. Intra-observer agreement demonstrated strong ICC values between 0.876 and 0.999, while inter-observer agreement ranged from 0.76 to 0.989, highlighting its reliability^14^.

#### Assessment of transverse abdominal muscle ratio

Utilizing a 5 MHz curvilinear transducer, ultrasound images were captured with the subjects in a supine position. The transducer's placement in a transverse plane occurred midway between the anterior superior iliac spine and the lower ribcage, along the anterior axillary line. The TrA activation ratio was measured in both resting and activation states for all patients, demonstrating high intra-observer agreement (ICC ranging from 0.95 to 1.00)^14^.

#### Assessment of multifidus muscles ratio

The measurement of multifus muscle thickness at the L4–L5 level was carried out in two distinct positions: static and dynamic. In the static position, the patient assumed a prone lying posture with a pillow beneath the abdomen to reduce lumbar lordosis. During this position, the patient was instructed to lift the contralateral arm, while in the dynamic position, contraction was induced. The dynamic position involved raising the upper body approximately 5 cm off the table. Additionally, the patient's upper limbs were positioned overhead, with elbows flexed at 90° and shoulders abducted to 120°^15^.

#### The Female Sexual Function Index (FSFI)

It is a 19-item questionnaire that assesses sexual function and problems. This assessment encompasses six aspects of female sexual function: desire, arousal, lubrication, pain, orgasm, and satisfaction during sexual activity within the preceding month. Each domain comprises 2–3 questions and holds a specific coefficient (0.6 for desire, 0.3 for arousal and lubrication, and 0.4 for orgasm, satisfaction, and pain), employed to compute the final domain score. The cumulative sum of individual domain scores yields a total score, where higher scores denote improved or more typical sexual function. The scoring system ranges from 2 to 36, with scores surpassing 26.5 indicating a satisfactory sexual life, while scores below 26.5 suggest compromised sexual function^16^. Arabic FSFI reliability (r from 0.92 to 0.98), high internal consistency (α from 0.85 to 0.94) and showed an excellent overall performance (area under the curve [AUC] = 0.985, 95% confidence interval 0.978–0.992)^17,18^.

#### Vaginal Laxity Questionnaires (VLQ)

Assesses the degree of vaginal laxity and tightness through a 7-point scale, ranging from 1 to 7. The scale includes the following descriptors: 1 for "very loose", 2 for "moderately loose", 3 for "slightly loose", 4 for "neither loose nor tight", 5 for "slightly tight", 6 for "moderately tight", and 7 for "very tight"^19^.

### Ethical approval

All procedures performed in studies involving human participants were by the ethical standards of the institutional and/or national research committee and with the 1964 Helsinki Declaration and its later amendments or comparable ethical standards. This study was designed as an observational and cross-sectional study and was approved by the Ethical Committee at Deraya University, El-Minya, Egypt (No: 6/2023). The study adhered to the ethical standards outlined in the Declaration of Helsinki and complied with principles for human research. All patients provided written consent after receiving a thorough description of the trial. The study was conducted at an outpatient clinic between February 1, 2023, and April 15, 2023. The clinical trial identifier for this study is NCT/05833685.

### Consent statement

Informed consent was obtained from all individual participants included in the study.

## Related work

Liu et al. ^20^ conducted a study to assess various machine learning approaches for predicting erectile dysfunction (ED) and analyzing the importance of ED risk factors. The investigated methods included logistic regression, multilayer feedforward backpropagation neural networks, fuzzy K-nearest neighbor classifier, support vector machine (SVM), and conventional discriminant function analysis. The results showed that the artificial neural network method achieved the highest ROC-AUC, indicating its superiority in developing a reliable model for predicting ED compared to the other models examined.

Li et al.^21^ conducted a study aiming to investigate cerebral structural changes associated with venous erectile dysfunction (VED), their correlation with clinical symptoms and disorder duration, and the use of machine learning to distinguish VED patients from healthy controls. The study included 45 VED patients and 50 healthy controls, utilizing voxel-based morphometry (VBM), tract-based spatial statistics (TBSS), and correlation analyses. VED patients exhibited decreased cortical volumes in specific brain regions and increased cortical volume in the right middle temporal gyrus. Widespread alterations in white matter microstructure were observed, with certain regions correlating with clinical symptoms and disorder duration. Machine learning analysis achieved an overall accuracy of 96.7%, sensitivity of 93.3%, and specificity of 99.0%, indicating the potential of DTI-derived indices as reliable discriminating features between VED patients and healthy controls.

Xu et al.^22^ conducted a study to investigate alterations in resting-state whole brain functional connectivity (FC) in lifelong premature ejaculation (LPE) patients. Utilizing a supported vector machine-based classification model with FC as features, the study aimed to identify specific FC patterns distinguishing LPE patients from healthy controls. The classification model achieved an accuracy of 0.85 ± 0.14, sensitivity of 0.92 ± 0.18, specificity of 0.72 ± 0.30, and recall index of 0.85 ± 0.17 across 1000 testing groups (100 times 10-folds cross-validation). Subsequent analyses identified four significant FCs, providing insight into abnormal central functional targets in LPE etiology. These findings, particularly the FC between bilateral medial parts of the orbital frontal cortex, suggest potential avenues for future interventions in LPE treatment.

In a study conducted by Liu et al.^23^, the prevalence of sexual dysfunction (SD) in patients with mental health disorders was emphasized, acknowledging its significant impact on their quality of life. The research aimed to address the often-overlooked identification of SD in clinical practice by exploring the use of machine learning (ML) models to identify high-risk individuals based on known risk factors. The study involved 135 subjects from a mental health clinic, utilizing health records data, including age, sex, diagnoses, drug treatment, and the Arizona Sexual Experiences Scale (ASEX). The ML model successfully identified individual SD cases with a balanced accuracy of 0.736, demonstrating its potential to enhance SD screening in psychiatric clinical settings. Major depressive disorder and female sex were identified as risk factors, while attention deficit hyperactivity disorder emerged as a potential protective factor. This study provides a proof-of-concept for ML-based SD screening in psychiatric patients, offering a promising avenue to optimize treatment options and improve their overall quality of life.

Hady et al. ^1^ conducted a study addressing urinary incontinence (UI) and its correlation with pelvic floor dysfunction (FSD) in women. UI, characterized by uncontrolled urine leakage, is linked to pelvic floor muscle (PFM) activity, impacting trunk and lumbo-pelvic stability. Traditional manual measurements for assessing pelvic tilt and lumbar angle are time-consuming and variable. This research aimed to predict core muscle activity in multiparous women with FSD, specifically pelvic tilt and lumbar angle, using decision tree, SVM, random forest, and AdaBoost models. The study achieved high accuracy, with AdaBoost performing best for pelvic tilt prediction (R^2^ = 0.944), and decision tree excelling for lumbar angle prediction (R^2^ = 0.976). The application of machine learning in predicting these parameters presents a potential revolution in the assessment and management of UI and FSD, offering faster, more accurate, and objective evaluations compared to traditional methods.

Machine learning has become increasingly popular in recent years for predicting and stratifying diseases that involve multiple factors^24^. By analyzing multiple variables, machine learning can identify important combinations for diagnosing and prognosing diseases^25^, and can detect nonlinear relationships between them^26^. This makes it a flexible tool for handling various types of variables and extracting hidden patterns that may not be visible to clinicians^27^. With the ability to handle large amounts of data, machine learning can achieve diagnostic accuracy comparable to or even better than that of clinicians^28^. Additionally, it has the potential to uncover insights that clinicians may not have noticed^29,30^. Unlike traditional methods that rely on established principles, machine learning relies on data to make predictions and stratifications. However, this approach also has its limitations as it can be prone to biases present in the data and may lack reproducibility. Therefore, it is important to exercise caution when using machine learning techniques and ensure that the data used for training and testing is representative and unbiased^31^. Additionally, methods for validating and reproducing the results should be carefully considered to ensure that the predictions made by the machine learning models are reliable and can be reproduced consistently^32^. The differences between machine learning and deep learning presented in Table 1 can be summarized as follows:

**Table 1:** The differences between machine learning and deep.

Criteria	Machine learning	Deep learning
Approach to learning	Based on statistical algorithms and models	Based on artificial neural networks
Dataset size	Typically used for smaller datasets	Particularly suited for processing large datasets
Data type	Can handle both structured and unstructured data	Best suited for unstructured data, such as images, audio, and text
Feature engineering	Requires feature engineering, or the manual selection and extraction of relevant features from the data	Can automatically learn relevant features through multiple layers of neural networks
Hardware requirements	Can be trained on a CPU	Requires specialized hardware, such as GPUs, for training and inference
Training time	Generally faster to train than deep learning models	Deep learning models can take longer to train due to the increased complexity of the neural networks
Applications	Can be used for a wide range of applications, including classification, regression, and clustering	Primarily used for applications such as image recognition, speech recognition, and natural language processing
Data requirements	Generally requires less data to achieve good performance	Requires large amounts of data to achieve good performance
Interpretability	Tends to be more interpretable, as the models are often based on simpler algorithms	Can be less interpretable, as the models can be highly complex and difficult to understand
Model size	Can work well with small to medium-sized models	Can handle very large models with many layers
Efficiency	Can be more efficient in terms of memory and computational requirements	Can be more memory-intensive and computationally expensive
Performance	Can achieve good performance even with less complex models	Can achieve state-of-the-art performance with highly complex models

These are general differences between the two approaches, and there may be specific cases where one approach is more appropriate than the other, depending on the problem at hand.

## Methodology

The proposed framework consists of the following steps:

### Data collection

A dataset comprising TrA ratio, MF ratio, Diaphragm, and PFM force measurements for women with incontinence and sexual dysfunction was collected from medical clinics and institutions. The dataset included demographic information, such as age, weight, height, and medical history.

### Feature selection

Statistical and machine learning techniques were employed to identify the most important features for predicting changes in TrA ratio, MF ratio, Diaphragm, and PFM force in cases of incontinence and sexual dysfunction. These features encompassed demographic information, medical history, and other pertinent factors.

### Machine learning algorithms

Multiple machine-learning algorithms, namely logistic regression, decision trees, random forests, and neural networks, were utilized for training and testing the models. The performance assessment of each algorithm was conducted through cross-validation techniques.

### Model training

The machine learning algorithms were trained on the gathered data using cross-validation techniques. The training data was split into training and testing sets to assess the performance of each algorithm.

### Model evaluation

The trained models were evaluated based on their accuracy, sensitivity, specificity, and other relevant metrics. A comparison was made to determine which algorithm was the most effective for predicting TrA ratio, MF ratio, Diaphragm, and PFM force changes in cases of incontinence and sexual dysfunction.

### Implementation

Once the most effective machine learning algorithm had been identified, it was implemented in a user-friendly interface for healthcare professionals to use in diagnosing and treating patients with incontinence and sexual dysfunction.

### Results

This project was expected to result in the development of a machine learning algorithm that could accurately predict changes in TrA ratio, MF ratio, Diaphragm, and PFM force in cases of incontinence and sexual dysfunction. The algorithm was intended to assist healthcare professionals in developing more effective treatment plans for patients with these conditions, ultimately aiming to improve their quality of life.

## Preliminaries

### Deep learning techniques

#### Multi-layer perceptron (MLP)

**Figure 1:**
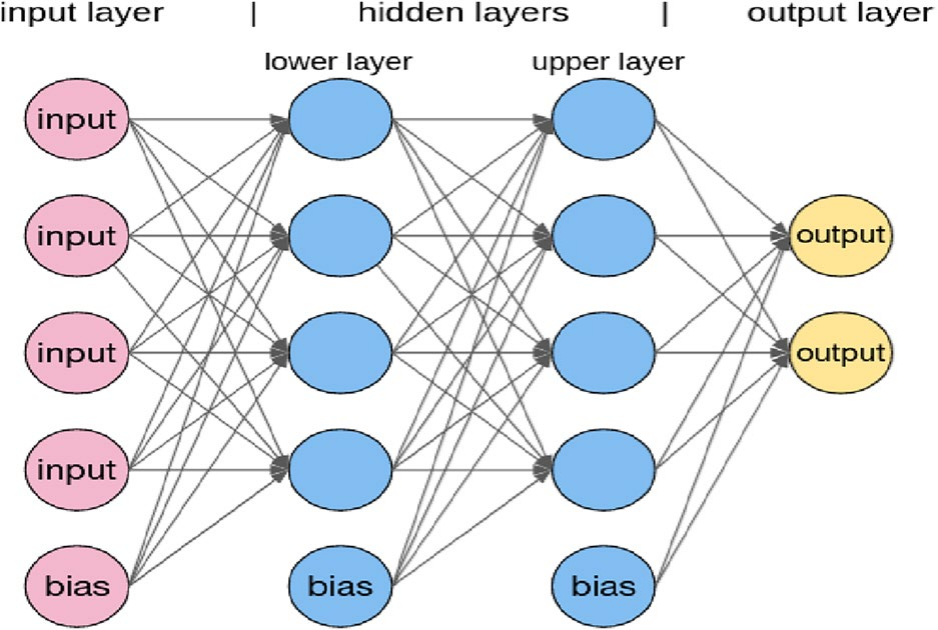
The architecture of the MLP.

MLP, which stands for multilayer perceptron, is a fundamental concept in the field of artificial neural networks and machine learning. It is one of the simplest and most widely used types of neural networks, known for its ability to solve a wide variety of problems, including classification, regression, and pattern recognition. At its core, an MLP is a feedforward neural network consisting of multiple layers of artificial neurons, or perceptrons, organized in a sequential manner. Each perceptron takes a set of inputs, applies a linear transformation on them, and then passes the transformed inputs through an activation function. The output of one layer serves as the input to the next layer until the final layer, which produces the network's output. The architecture of an MLP typically consists of an input layer, one or more hidden layers, and an output layer as shown in Fig. 1^33^. The input layer simply receives the input data, while the hidden layers perform intermediate computations, and the output layer generates the final predictions or classifications^34^.

The key characteristic of an MLP is its ability to learn and adapt to complex relationships between inputs and outputs through a process called training. During training, the network adjusts the weights and biases associated with each perceptron based on the error or loss between its predictions and the desired outputs. This adjustment is done using optimization algorithms like gradient descent, which iteratively updates the network's parameters to minimize the loss. MLPs are known for their capability to model nonlinear relationships thanks to the activation functions used in each perceptron. Commonly used activation functions include the sigmoid function, hyperbolic tangent function, and rectified linear unit (ReLU) function. These nonlinear functions introduce nonlinearity into the network, enabling it to capture and represent complex patterns in the data^35^.

## Long short-term memory (LSTM)

**Figure 2:**
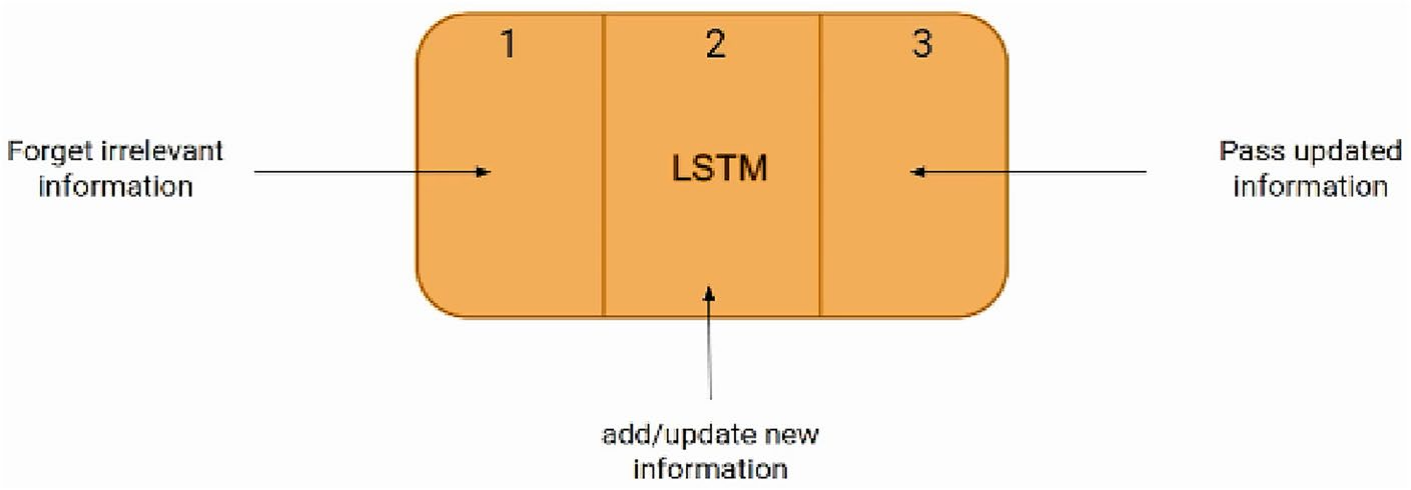
The architecture of the LSTM.

Long short-term memory (LSTM) is a specialized type of recurrent neural network (RNN) architecture renowned for efficiently handling sequences and temporal dependencies. Unlike standard feed-forward networks incapable of retaining historical memory, LSTMs possess cell states encapsulating long-range contextual information, empowering them to maintain intricate sequence representations. An LSTM unit comprises three gate structures—input, forget, and output gates—governing cell state manipulations at every timestep as shown in Fig. 2^36^. Precisely controlling inflow, discarding irrelevant details, and strategically releasing pertinent clues enables LSTMs to thrive in environments typified by vanishing gradients, afflicting regular RNNs attempting lengthier dependency exploitations^37^.

Input gate activation determines whether fresh input warrants integration into the cell state, guided by multiplicative interaction alongside candidate values generated from tanh operations. Meanwhile, the forget gate weighs significance attached to prevailing cell contents, deciding proportions consigned to oblivion or retained following gating mechanism enforcement. Lastly, the output gate governs disclosure magnitude directed to subsequent cells or external entities, contingent upon current cell state appraisals. Impressive achievements materialize courtesy of LSTMs in domains necessitating extended memory preservation, notably sentiment analysis, music generation, and speech synthesis. Beyond mere symbolic series modeling, LSTMs demonstrate striking aptitude in extracting latent semantic attributes concealed beneath surface appearances, substantially bolstering predictive performance.

## Convolutional neural network (CNN)

Convolutional neural network (CNN) is a type of deep neural network that is designed to process images and videos. CNNs are particularly effective at automatically learning and identifying features in images, which are then used to make predictions. CNNs consist of multiple layers, including convolutional layers, pooling layers, and fully connected layers. The convolutional layers perform feature extraction by applying convolutional filters to the input image. The pooling layers reduce the dimensionality of the feature maps produced by the convolutional layers. The fully connected layers process the output from the pooling layers to produce the final predictions. CNNs have been successfully applied in various domains, including object detection, facial recognition, and medical image analysis. CNNs are powerful deep-learning algorithms that can handle complex image data with high accuracy^26,38^.

## Recurrent neural network (RNN)

Recurrent neural network (RNN) is a type of neural network that is designed to handle sequential data, such as time-series data or natural language processing. RNNs are particularly effective in processing sequential data by maintaining a memory of previous inputs and using that memory to make predictions about the current input. RNNs consist of recurrent layers, which allow information to be passed from one-time step to the next. The input at each time step is processed by the recurrent layer, and the output is used to update the state of the layer. This state is then passed to the next time step, allowing the network to maintain a memory of previous inputs. RNNs have been successfully applied in various domains, including speech recognition, language modeling, and machine translation. RNNs are powerful deep-learning algorithms that can handle complex sequential data with variable-length inputs^39^.

### Machine learning techniques

### ElasticNetCV

ElasticNetCV is a type of linear regression model that combines the Lasso and Ridge regression techniques. ElasticNetCV is used for regression tasks where the number of features is much larger than the number of samples. ElasticNetCV adds a regularization term to the loss function to prevent overfitting and to help the model generalize better to new data. ElasticNetCV uses cross-validation to find the optimal values of the hyperparameters alpha and l1_ratio. ElasticNetCV has been successfully applied in various domains, including finance, healthcare, and energy^40^.

## Random forest regressor

RandomForestRegressor is a type of ensemble learning algorithm that combines multiple decision trees to make predictions. RandomForestRegressor is used for regression tasks where the input data has high dimensionality and is non-linear. RandomForestRegressor randomly selects a subset of features and samples from the input data to train each decision tree. The predictions of the individual decision trees are then combined to produce the final prediction. RandomForestRegressor is robust to overfitting and can handle missing data. RandomForestRegressor has been successfully applied in various domains, including finance, healthcare, and marketing^41,42^.

### SVR

Support vector regression (SVR) is a type of regression algorithm that is based on the support vector machine (SVM) algorithm. SVR is used for regression tasks where the input data has non-linear relationships. SVR maps the input data to a high-dimensional feature space and constructs a hyperplane that maximizes the margin between the predicted values and the actual values. SVR uses a kernel function to transform the input data into a higher-dimensional space, where it is easier to separate the classes. SVR has been successfully applied in various domains, including finance, healthcare, and engineering^43^.

## Bagging regressor

BaggingRegressor is a type of ensemble learning algorithm that combines multiple regression models to make predictions. BaggingRegressor is used for regression tasks where the input data has high variability and is non-linear. BaggingRegressor randomly selects a subset of features and samples from the input data to train each regression model. The predictions of the individual regression models are then combined to produce the final prediction. BaggingRegressor is robust to overfitting and can handle missing data. BaggingRegressor has been successfully applied in various domains, including finance, healthcare, and marketing^44^.

## The proposed framework

**Figure 3:**
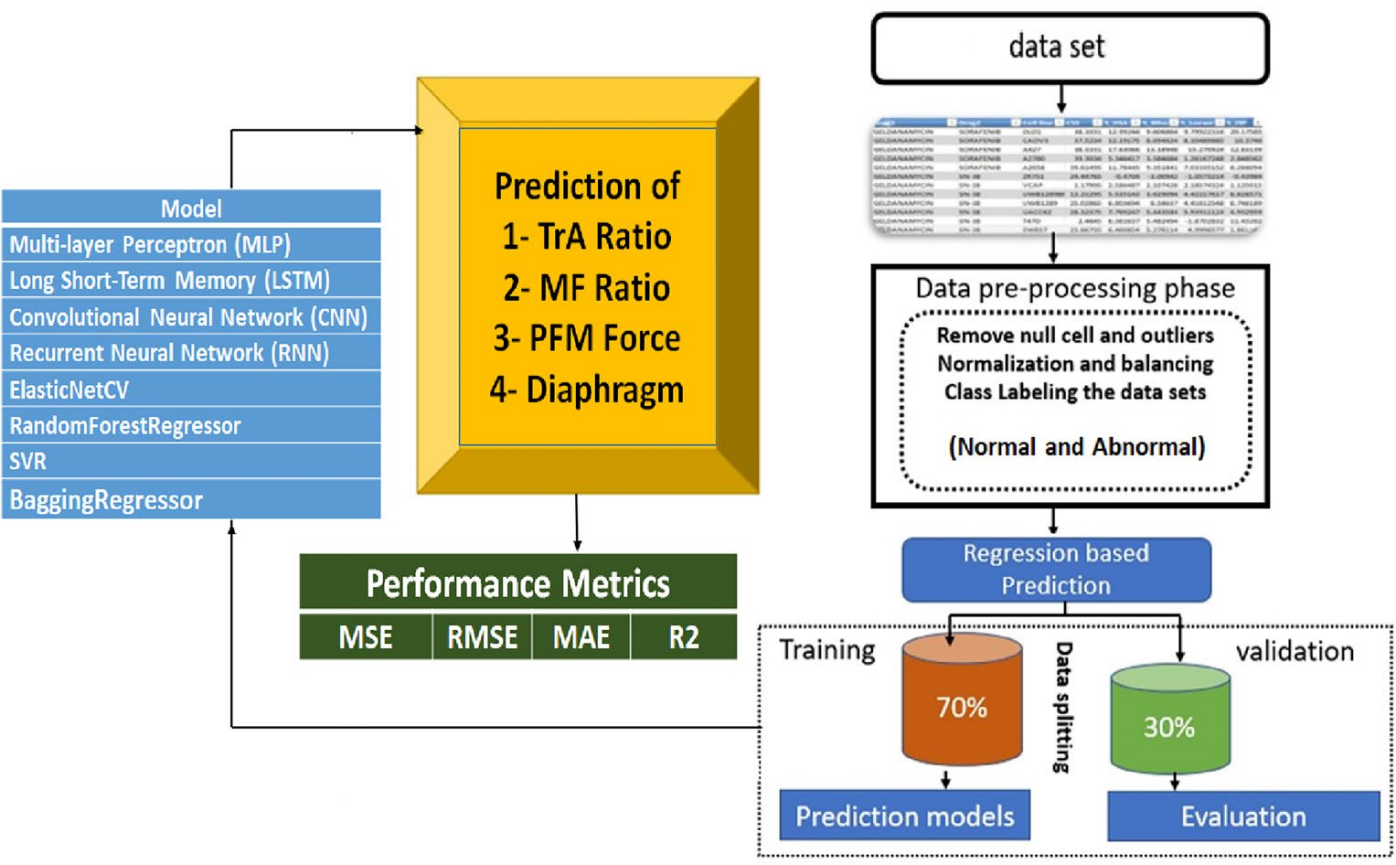
The general framework of the proposed prediction model.

We designed a machine-learning framework to identify the values of the TrA ratio, MF ratio, PFM force, and Diaphram. Figure 3 investigates the general structure of the proposed framework and demonstrates the prediction process and the performance metrics.

### Dataset characteristics

The characteristics of the dataset can be described as follows:

- TrA R: the strength or activation level of the right transverse abdominal muscle, which is a core muscle.
- TrA c: the strength or activation level of the left transverse abdominal muscle, which is a core muscle.
- TrA ratio: the ratio of strength or activation level between the right and left transverse abdominal muscles.
- MF ratio: the ratio of strength or activation level between the multifidus muscles, which are deep muscles in the back that help support the spine.
- MF R: the resting activity level of the multifidus muscles.
- MF CONT: the continuous activity level of the multifidus muscles.
- Diaphragm: diaphragm muscle, which is a muscle involved in breathing.
- PFM Force: the strength or activation level of the pelvic floor muscles.
- FSFI: Female Sexual Function Index, a questionnaire used to assess sexual function in women.
- VLQ: Vestibular Labyrinthine Questionnaire, a questionnaire used to assess vestibular function.
- Age: the age of the individual.
- Weight: the weight of the individual in kilograms.
- Height: the height of the individual in centimeters.
- BMI: Body Mass Index, a measure of body fat based on height and weight.
- Status: this could refer to the overall health or functional status of the individuals being measured (Normal or Abnormal).

**Figure 4:**
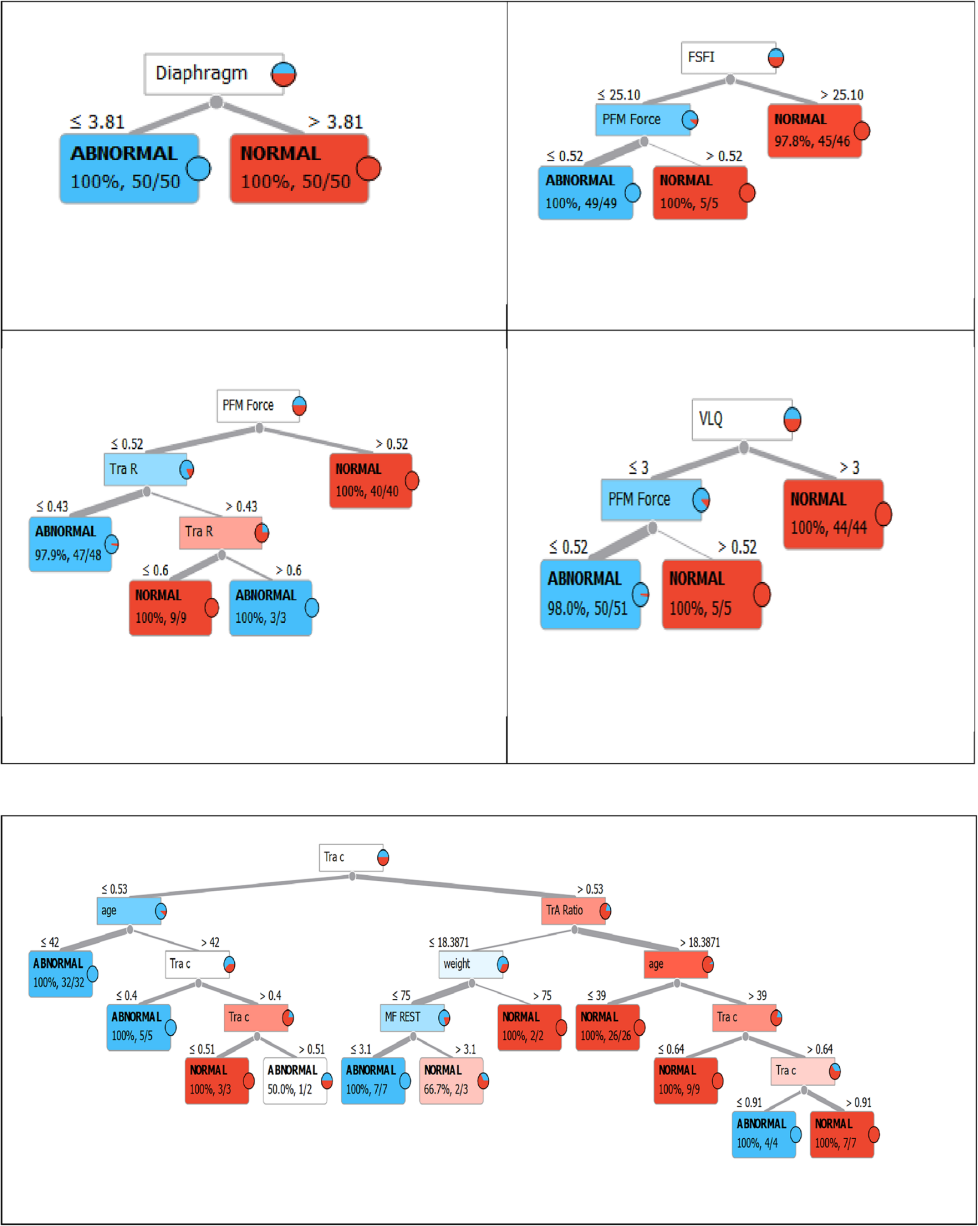
The relationships between the variables Tra R, Tra c, TrA ratio, MF ratio, MF REST, MF CONT, Diaphragm, PFM Force, FSFI, and VLQ of females with sexual dysfunction.

Figure 4 displays the relationships between the variables Tra R, Tra c, TrA ratio, MF ratio, MF REST, MF CONT, Diaphragm, PFM force, FSFI, and VLQ, which are the features used in the study.

**Figure 5:**
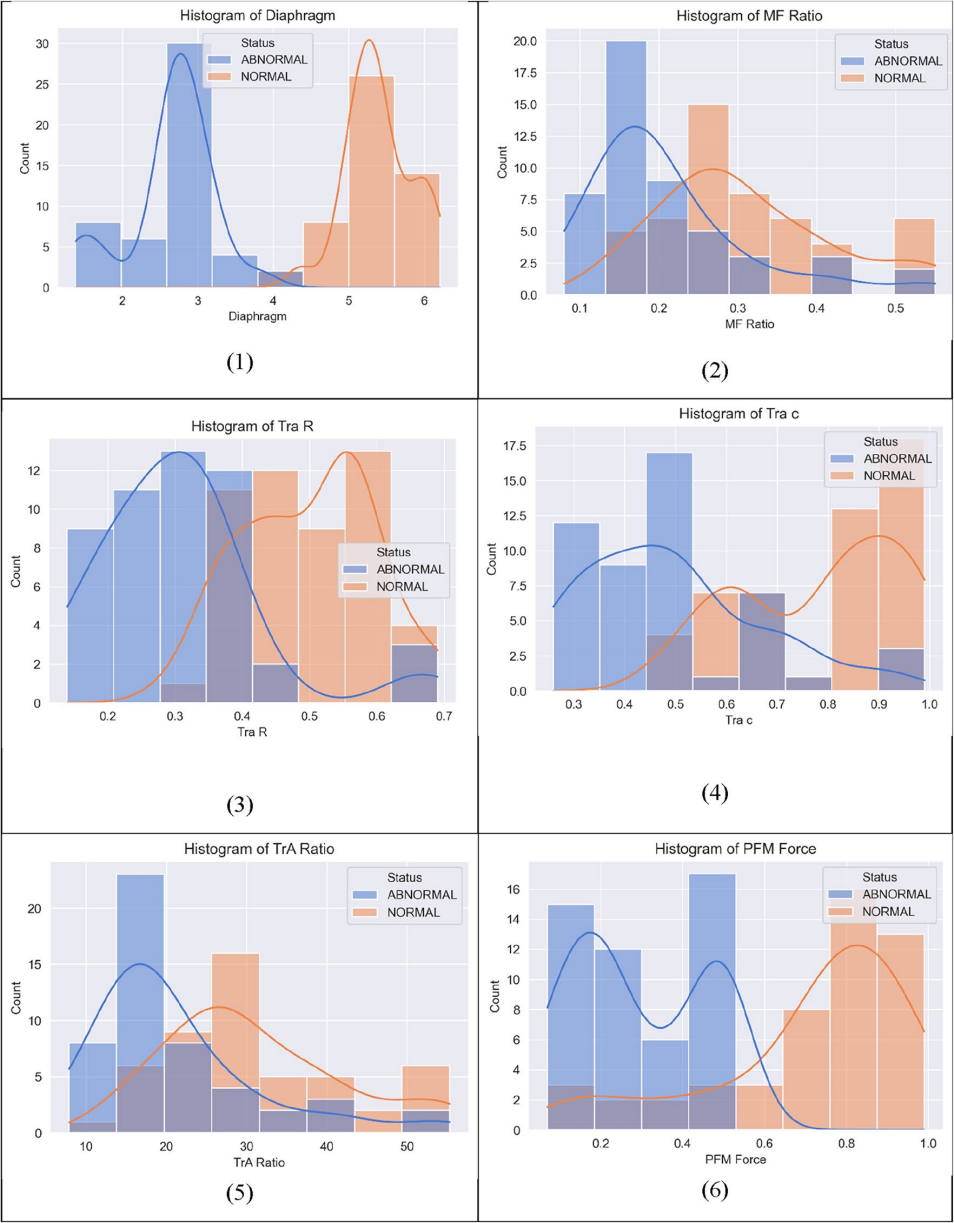
Correlation between Tra Rest, Tra c, TrA ratio, MF ratio, Diaphragm, and PFM force.

Figure 5 shows the correlation between the lumbar angle and pelvic tilt in two groups of women: normal females and females with sexual dysfunction, specifically urinary incontinence (UI).

The dataset's numerical variable correlation is shown in Table 2. Each row and column in the correlation matrix represents a continuous variable, and each value indicates the correlation coefficient (Pearson's R-value) between the variables represented by that row and column. Most attributes are highly correlated, according to our observations. This is a correlation matrix that describes the relationships between different attributes. Each attribute is listed on both the rows and columns. The values in the cells represent the correlation coefficient between the two attributes. A correlation coefficient close to 1 indicates a strong positive correlation, while a coefficient close to − 1 indicates a strong negative correlation. A coefficient close to 0 indicates no correlation.

**Table 2:** The correlation heat map of the proposed framework.

	Tra R	Tra c	TrA Ratio	MF Ratio	MF REST	MF CONT	Diaphram	PFM Force	FSFI	VLQ	age	weight	height	BMI
Tra R	1.00	0.89	0.22	0.21	0.28	0.31	0.62	0.41	0.56	0.56	− 0.05	− 0.11	− 0.01	− 0.06
Tra c	0.89	1.00	0.27	0.27	0.34	0.38	0.64	0.45	0.55	0.53	− 0.09	− 0.10	0.04	− 0.09
TrA ratio	0.22	0.27	1.00	1.00	0.36	0.70	0.47	0.33	0.35	0.39	− 0.16	0.01	0.05	− 0.02
MF ratio	0.21	0.27	1.00	1.00	0.36	0.69	0.47	0.33	0.35	0.38	− 0.16	0.01	0.06	− 0.02
MF REST	0.28	0.34	0.36	0.36	1.00	0.92	0.30	0.18	0.39	0.23	− 0.13	− 0.04	− 0.07	0.04
MF CONT	0.31	0.38	0.70	0.69	0.92	1.00	0.44	0.28	0.44	0.34	− 0.18	− 0.03	− 0.04	0.02
Diaphram	0.62	0.64	0.47	0.47	0.30	0.44	1.00	0.69	0.73	0.82	− 0.10	− 0.05	0.12	− 0.12
PFM force	0.41	0.45	0.33	0.33	0.18	0.28	0.69	1.00	0.43	0.54	− 0.05	− 0.13	0.15	− 0.19
FSFI	0.56	0.55	0.35	0.35	0.39	0.44	0.73	0.43	1.00	0.74	0.10	0.03	0.01	0.01
VLQ	0.56	0.53	0.39	0.38	0.23	0.34	0.82	0.54	0.74	1.00	0.05	0.00	0.06	− 0.04
Age	− 0.05	− 0.09	− 0.16	− 0.16	− 0.13	− 0.18	− 0.10	− 0.05	0.10	0.05	1.00	0.07	0.01	0.04
Weight	− 0.11	− 0.10	0.01	0.01	− 0.04	− 0.03	− 0.05	− 0.13	0.03	0.00	0.07	1.00	0.06	0.66
Height	− 0.01	0.04	0.05	0.06	− 0.07	− 0.04	0.12	0.15	0.01	0.06	0.01	0.06	1.00	− 0.71
BMI	− 0.06	− 0.09	− 0.02	− 0.02	0.04	0.02	− 0.12	− 0.19	0.01	− 0.04	0.04	0.66	− 0.71	1.00

### Data preprocessing

Data preprocessing refers to the steps taken to prepare the raw data for machine learning algorithms. These steps are important as they can greatly affect the accuracy and performance of the model. Some common data preprocessing steps are:

- Data cleaning: this involves removing any noise or outliers in the data, filling in missing values, and correcting any inconsistencies or errors in the data.
- Data transformation: this involves converting the data into a suitable format for the machine learning algorithms. For example, converting categorical data into numerical data, and normalizing or standardizing the data.
- Feature engineering: this involves selecting or creating the most relevant features or variables for the model. This can involve feature selection, dimensionality reduction, and creating new features based on domain knowledge.
- Data splitting: this involves splitting the data into training, validation, and test sets. The training set is used to train the model, the validation set is used to tune the hyperparameters, and the test set is used to evaluate the model's performance on unseen data.
- Data augmentation: this involves artificially increasing the size of the dataset by creating variations of the existing data. This can be useful for improving the model's robustness and generalization.
- These steps are iterative and may need to be repeated multiple times depending on the quality and complexity of the data. The goal is to prepare a clean and relevant dataset that will allow the machine learning algorithm to learn and make accurate predictions.

### Evaluation metrics for regression models

The determination coefficient R-square is one of the most common performances used to evaluate the regression model as shown in Eq. (1). On the other hand, the Minimum Acceptable Error (MAE) is shown in Eq. (2), while the Mean Square Error (MSE) is investigated in Eq. (3)^45^.

1 $${{\text{R}}}^{2}=\frac{\sum {\left(y-\dot{\widehat{y}}\right)}^{2}}{\sum {\left(y-\dot{\overline{y}}\right)}^{2}}$$

2 $${\text{MAE}}=\frac{\sum_{i=1}^{n}\left|\widehat{{y}_{i}}-y\right|}{{\text{n}}}$$

3 $${\text{MSE}}=\frac{\sum_{i=1}^{n}\left|\widehat{{y}_{i}}-{y}_{i}\right|}{{\text{n}}}$$

where y is the actual value, $\dot{\widehat{{\text{y}}}}$ is the corresponding predicted value, $\dot{\overline{{\text{y}}}}$ is the mean of the actual values in the set, and ***n*** is the total number of test objects^31,46^.

## Results and analysis

In this section, we have conducted experiments to assess the performance of the machine learning framework for predicting the TrA ration, the MF ratio, the PFM force and the diaphragm excursion. We are conducting our experiments on a 3 GHz i5 computer with an 8 GB main memory and 64-bit Windows 10 operating system. The experiment is carried out using the Python programming language.

### Predicting the TrA ratio using regression machine learning techniques

**Figure 6:**
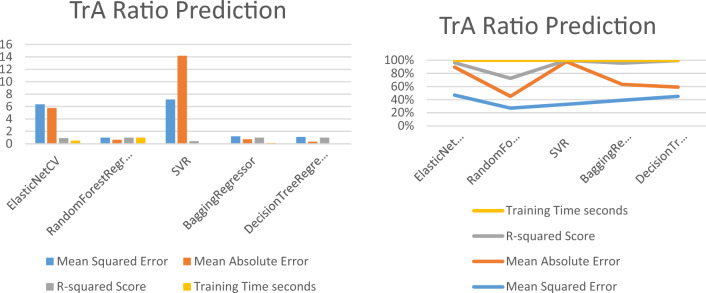
The performance metrics of five different regression models to predict TrA ratio.

Table 3 and Fig. 6 display the performance metrics of five different regression models, including ElasticNetCV, random forest regressor, SVR, Bagging regressor, and decision tree regressor. The table presents the mean squared error (MSE), mean absolute error (MAE), and R-squared Score, which are commonly used metrics to evaluate the accuracy and precision of regression models. Additionally, the table shows the training time for each model, which is the time taken by the model to fit the training data.

**Table 3:** The performance metrics of five different regression models to predict the TrA ratio.

Model	Mean squared error	Mean absolute error	R-squared score	Training time (s)
ElasticNetCV	6.3584	5.7591	0.9152	0.5107
RandomForestRegressor	0.9912	0.6601	0.9982	1.0054
SVR	7.1506	14.1606	0.4323	0.0250
BaggingRegressor	1.2072	0.7490	0.9979	0.1359
DecisionTreeRegressor	1.1100	0.3500	0.9979	0.0110

Table 3 shows that the random forest regressor model outperforms the other models, with the lowest MSE and MAE, and the highest R-squared Score. However, it has a longer training time compared to the other models. The Bagging regressor and decision tree regressor models also perform well, with comparable performance metrics and lower training times. The SVR model has the highest MSE and MAE, and the lowest R-squared Score, indicating that it may not be the best model for this dataset. The ElasticNetCV model also has a relatively high MSE and MAE, indicating that it may not perform as well as the other models.

### Predicting the MF ratio using regression machine learning techniques

**Figure 7:**
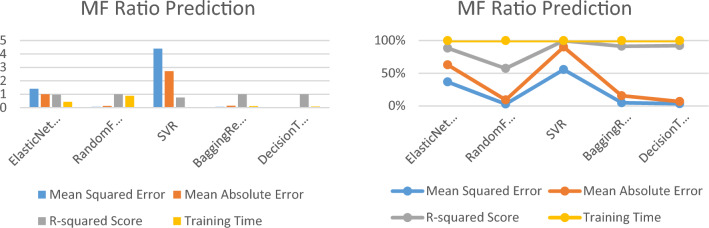
Performance metrics of the MF ratio prediction models.

Table 4 and Fig. 7 present the performance metrics of five different regression models, including ElasticNetCV, random forest regressor, SVR, Bagging regressor, and decision tree regressor. The table shows the mean squared error (MSE), mean absolute error (MAE), and R-squared Score, which are commonly used to evaluate the accuracy and precision of regression models. Additionally, the table shows the training time for each model, which is the time taken by the model to fit the training data.

**Table 4:** The performance metrics of five different regression models to predict MF ratio.

Model/MF ratio	Mean squared error	Mean absolute error	R-squared score	Training time (s)
ElasticNetCV	1.4063	0.9998	0.9755	0.4397
RandomForestRegressor	0.0614	0.1303	0.9990	0.8826
SVR	4.4012	2.7196	0.7579	0.0220
BaggingRegressor	0.0643	0.1430	0.9989	0.1149
DecisionTreeRegressor	0.0400	0.0400	0.9994	0.090

Figure 7 and Table 4 provide a clear comparison of the performance of each model, allowing for easy evaluation of their accuracy and precision. The random forest regressor model performs the best with the lowest MSE and MAE, and a high R-squared Score. The decision tree regressor model also performs well, with the lowest MAE and MSE, and a high R-squared Score. The Bagging regressor model performs similarly to the random forest regressor model, with comparable performance metrics and a shorter training time. The ElasticNetCV model and the SVR model have higher MSE and MAE values, indicating that they may not perform as well as the other models for this particular dataset.

### Predicting the PFM force using regression machine learning techniques

**Figure 8:**
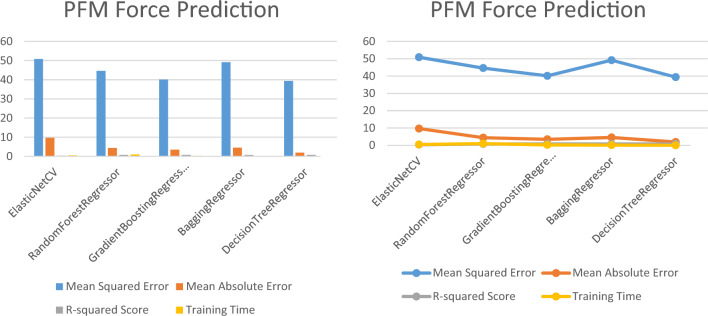
Performance metrics of the PFM force prediction models.

Table 5 and Fig. 8 show the performance metrics of five different regression models, including ElasticNetCV, random forest regressor, SVR, Bagging regressor, and decision tree regressor, for the feature MF ratio. The table presents the mean squared error (MSE), mean absolute error (MAE), and R-squared Score, which are commonly used to evaluate the accuracy and precision of regression models. Additionally, the table shows the training time for each model, which is the time taken by the model to fit the training data.

**Table 5:** The performance metrics of five different regression models to predict PFM force.

Model/PFM force	Mean squared error	Mean absolute error	R-squared score	Training time (s)
ElasticNetCV	50.8448	9.6778	0.3830	0.4897
RandomForestRegressor	44.5879	4.4092	0.7928	0.9864
GradientBoostingRegressor	40.0984	3.4776	0.8146	0.2901
BaggingRegressor	49.1420	4.5620	0.7724	0.1239
DecisionTreeRegressor	39.3700	1.9300	0.8089	0.0100

Table 5 and Fig. 8 provide a comparison of the performance of each model for the PFM force feature, allowing for easy evaluation of their accuracy and precision. The gradient boosting regressor model performs the best with the lowest MSE and MAE, and a high R-squared Score. The random forest regressor model and the decision tree regressor model also perform well, with high R-squared Scores and low MAE and MSE values. The Bagging regressor model and the ElasticNetCV model have relatively high MSE and MAE values, indicating that they may not perform as well as the other models for this particular feature.

### Predicting the diaphragm excursion using regression machine learning techniques

**Figure 9:**
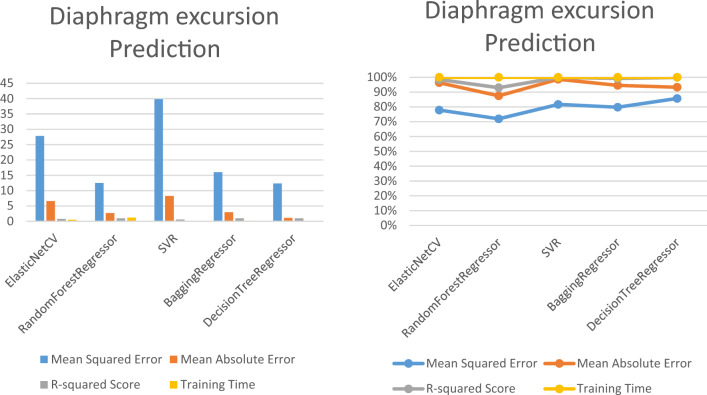
Performance metrics of the diaphragm excursion prediction models.

Table 6 and Fig. 9 present the performance metrics of five different regression models, including ElasticNetCV, random forest regressor, SVR, Bagging regressor, and decision tree regressor, for the feature Diaphragm. The table displays the mean squared error (MSE), mean absolute error (MAE), and R-squared Score, which are commonly used to evaluate the accuracy and precision of regression models. Additionally, the table shows the training time for each model, which is the time taken by the model to fit the training data.

**Table 6:** The performance metrics of five different regression models to predict diaphragm excursion.

Model/diaphragm excursion	Mean squared error	Mean absolute error	R-squared score	Training time (s)
ElasticNetCV	27.8432	6.6115	0.7701	0.5457
RandomForestRegressor	12.5071	2.7001	0.9550	1.2257
SVR	39.8218	8.2794	0.6164	0.0340
BaggingRegressor	16.0264	2.9615	0.9426	0.1589
DecisionTreeRegressor	12.3300	1.0900	0.9544	0.0140

It appears that the decision tree regressor and random forest regressor models perform the best in terms of their low MSE and high R-squared scores, but they are relatively slow to train compared to the other models. The SVR model is the fastest but has the worst performance in terms of MSE and R-squared. The ElasticNetCV and Bagging regressor models have relatively good performance and moderate training times. The choice of the best model would depend on the specific application and the tradeoff between accuracy and training time.

### Feature correlations and feature selection

Table 7 presents Pearson's correlation coefficients between various features. The correlation coefficient ranges from − 1 to 1, where a value of 1 indicates a perfect positive correlation between two features, a value of 0 indicates no correlation and a value of − 1 indicates a perfect negative correlation.

**Table 7:** Pearson’s correlation of the features.

First feature	Second feature	Correlation	First feature	Second feature	Correlation
TrA Ratio	MF Ratio	0.998	Age	PFM force	− 0.052
MF REST	MF CONT	0.909	Weight	MF REST	− 0.047
TrA c	TrA R	0.902	Weight	Diaphragm	− 0.045
VLQ	Diaphragm	0.774	BMI	Age	0.044
VLQ	FSFI	0.767	Age	VLQ	0.041
FSFI	Diaphragm	0.75	Weight	Age	0.034
Weight	BMI	0.719	Height	FSFI	0.032
MF Ratio	MF CONT	0.648	Weight	VLQ	− 0.023
TrA Ratio	MF CONT	0.64	BMI	TrA ratio	− 0.018
PFM Force	Diaphragm	0.634	Height	TrA R	− 0.018
TrA R	Diaphragm	0.6	Weight	MF CONT	− 0.017
TrA R	FSFI	0.595	Weight	FSFI	− 0.015
TrA c	Diaphragm	0.594	Height	Age	− 0.015

Table 7 shows high positive correlations between MF ratio and TrA ratio (0.998) and between MF CONT and MF REST (0.909), indicating that these features are highly related. The Diaphragm feature has moderate positive correlations with VLQ (0.774), FSFI (0.75), TrA R (0.6), TrA c (0.594), PFM Force (0.634), TrA Ratio (0.498), and MF Ratio (0.496). The BMI feature has moderate positive correlations with weight (0.719) and moderate negative correlations with height (− 0.567).

The table also shows moderate positive correlations between TrA ratio and Tra c (0.64), Tra R and VLQ (0.585), FSFI and Tra R (0.595), and Tra c and VLQ (0.53). Additionally, there are moderate positive correlations between PFM force and Tra c (0.436), PFM force and Tra R (0.427), and between MF CONT and PFM force (0.243).

Feature selection is a process used in machine learning to identify the most relevant and useful features from a set of features that are used to train a model. The goal of feature selection is to improve the accuracy and efficiency of the model by reducing the number of features used for training. Table 8 shows the results of various feature selection techniques and the most important features selected by each technique. The table lists five different techniques, including F-value selector, mutual information selector, RFE with logistic regression, Selection from the model with random forests, and variance thresholding.

**Table 8:** Feature selection techniques and the most important features.

Technique	Most important features
F-value selector	(['TrA c', 'Diaphragm', 'PFM Force', 'FSFI', 'VLQ'])
Mutual information selector	(['TrA c', 'Diaphragm', 'PFM Force', 'FSFI', 'VLQ'])
RFE with logistic regression	(['Diaphragm', 'PFM Force', 'FSFI', 'VLQ', 'age'])
Select from the model with random forests	(['Diaphragm', 'PFM Force', 'FSFI', 'VLQ'])
Variance there holding	(['TrA Ratio', 'MF REST', 'MF CONT', 'Diaphragm', 'FSFI', 'VLQ', 'age', 'weight', 'height', 'BMI)

For the F-value selector and mutual information selector techniques, the most important features selected were 'TrA c', 'Diaphragm', 'PFM force', 'FSFI', and 'VLQ', indicating that these features are highly relevant for predicting the outcome variable. For the RFE with logistic regression technique, the most important features selected were 'Diaphragm', 'PFM force', 'FSFI', 'VLQ', and 'age', indicating that these features contribute significantly to the outcome variable and should be considered in a regression model.

The Select from the model with random forests technique selected 'Diaphragm', 'PFM force', 'FSFI', and 'VLQ' as the most important features. This indicates that these features have a high impact on the model's accuracy and should be included in a regression model. Finally, the variance thresholding technique selected 'TrA ratio', 'MF REST', 'MF CONT', 'Diaphragm', 'FSFI', 'VLQ', 'age', 'weight', 'height', and 'BMI' as the most important features, indicating that these features have a high variance and may have a significant impact on the outcome variable.

Table 8 provides an overview of the most important features selected by various feature selection techniques, which can help in selecting the best features for a regression model. The selected features can improve model accuracy, and the techniques can help in reducing the number of features, which can make the model more interpretable and efficient.

### A comparison among deep learning and classical machine learning regression techniques

**Figure 10:**
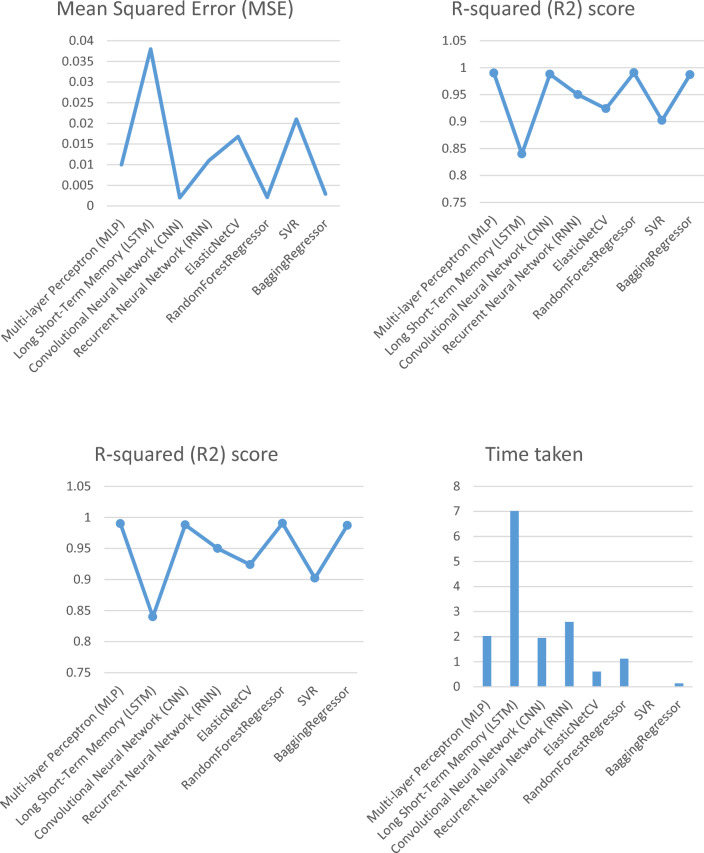
Evaluation metrics for machine and deep learning algorithms used to predict changes in core muscles during FSD.

The necessary libraries and modules are imported at the beginning of the script, including Pandas for data manipulation, NumPy for numerical computations, and Scikit-learn for data preprocessing and evaluation. The script also utilizes Keras, which is a high-level neural networks API written in Python and built on top of TensorFlow. The model architecture includes an input layer with the same number of neurons as the number of features in the input dataset, followed by two hidden layers with 32 and 16 neurons, respectively, and a final output layer with one neuron. The rectified linear unit (ReLU) activation function is used for all hidden layers, as it is known to perform well in deep learning models. The model is trained on the training set using Keras' fit method, with 50 epochs and a batch size of 64. The verbose argument is set to 0 to suppress progress output. Table 9 and Fig. 10 provide a summary of the evaluation metrics for different machine and deep learning algorithms used to predict changes in core muscles during FSD. The table includes the mean squared error (MSE), mean absolute error (MAE), R-squared (R^2^) score, and time taken by each algorithm.

**Table 9:** The evaluation metrics for different machine and deep learning algorithms.

Model	Mean squared error (MSE)	Mean absolute error (MAE)	R-squared (R^2^) score	Time taken
Deep learning algorithms				
Multi-layer perceptron (MLP)	0.01	0.03	0.99	2.03
Long short-term memory (LSTM)	0.038	0.12	0.84	7.016
Convolutional neural network (CNN)	0.002	0.030	0.988	1.95
Recurrent neural network (RNN)	0.011	0.057	0.95	2.59
Machine learning algorithms				
ElasticNetCV	0.0168	0.1107	0.9240	0.6094
RandomForestRegressor	0.0021	0.0117	0.9905	1.1250
SVR	0.0210	0.1146	0.9022	0.0156
BaggingRegressor	0.0029	0.0150	0.9871	0.1406

Four deep learning algorithms were used in the study: multi-layer perceptron (MLP), long short-term memory (LSTM), convolutional neural network (CNN), and recurrent neural network (RNN). The evaluation metrics show that CNN had the lowest MSE (0.002), followed by MLP (0.01), RNN (0.011), and LSTM (0.038). CNN also had the highest R^2^ score (0.988), indicating that it explained the most variance in the data. However, CNN took the longest time (1.95) among the deep learning algorithms. Four machine learning algorithms were also used in the study: ElasticNetCV, random forest regressor, SVR, and Bagging regressor. Random forest regressor had the lowest MSE (0.0021), followed by Bagging regressor (0.0029) and ElasticNetCV (0.0168). Random forest regressor also had the highest R^2^ score (0.9905) among the machine learning algorithms. The time taken by the machine learning algorithms was relatively low, with the highest time taken being 0.6094 by ElasticNetCV.

The evaluation metrics suggest that both deep learning and machine learning algorithms can be effective in predicting changes in core muscles during FSD. However, the choice of algorithm may depend on the specific use case and the trade-off between accuracy and time taken. For instance, if time is a crucial factor, machine learning algorithms such as random forest regressor or Bagging regressor may be more appropriate. On the other hand, if accuracy is the priority, deep learning algorithms such as CNN may be more suitable.

## Discussion and future directions

In this study, we developed and compared five different regression models to predict the TrA ratio, MF ratio, PFM force, and diaphragm excursion, utilizing various evaluation metrics, including mean squared error (MSE), mean absolute error (MAE), R-squared Score, and training time.

Our findings reveal that the random forest regressor outperformed other models in predicting the TrA ratio and MF Ratio, exhibiting the lowest MSE, MAE, and the highest R-squared Score among all models. Despite its slightly longer training time, random forest regressor proved to be the most accurate and precise model for these ratios. Similar results were observed for predicting PFM force, where the gradient boosting regressor demonstrated superior performance compared to the other models. However, when predicting diaphragm excursion, the decision tree regressor and random forest regressor models stood out with their low MSE and high R-squared scores, although their training times were slower than those of other models. The ElasticNetCV and Bagging regressor models had relatively good performance and moderate training times, making them reasonable options depending on the specific application and the tradeoff between accuracy and training time.

The Pearson correlation analysis exposed strong positive correlations between MF ratio and TrA ratio, as well as between MF CONT and MF REST. Additionally, the Diaphragm feature showed notable positive correlations with multiple features, including VLQ, FSFI, TrA R, TrA c, PFM force, TrA ratio, and MF ratio. These correlations emphasize the importance of considering these features together to gain deeper insights and improve the accuracy of predictive models. Feature selection techniques helped narrow down the most relevant features for the regression models. Among the top-selected features were 'TrA c', 'Diaphragm', 'PFM force', 'FSFI', and 'VLQ', which appeared consistently across various techniques. Integrating these features into the models resulted in improved accuracy and reduced complexity. Lastly, a comparison between deep learning and classical machine learning regression techniques indicated that deep learning algorithms might offer higher accuracy but demand more resources in terms of computing time and energy consumption. As such, balancing accuracy and efficiency remains a key challenge in determining the ideal model for predicting changes in core muscles during FSD.

This study put forward the following findings: showed a greater positive correlation between MF ratio and TrA ratio. The Diaphragm feature has moderate positive correlations with VLQ (0.774), FSFI (0.75), TrA R (0.6), TrA c (0.594), PFM force (0.634), TrA ratio (0.498), and MF ratio. The core can be described as a muscular box with the transverse abdominal in the front, multifidus in the back, the diaphragm as the roof, and the pelvic floor in the bottom, without these muscles, the spine would become mechanically unstable^47^. The multifidus muscles work with the transverses abdominal and the pelvic floor muscles to form what is known as the anatomical girdle, and when the abdominal muscles are powerfully contracted, the diaphragm goes higher and the elevated IAP causes a contraction of the PFM^48^. The pelvic floor consists of a bed of muscles underneath the pelvis, and it provides structural support for the internal organs, PFM strength in women is positively related to sexual function and arousal^49^. Another explanation, there is coordination between core muscles, the pelvic floor muscles (PFM), which are a part of IAP and respiration, are frequently disregarded. When abdominal muscles are contracted, the PFM goes downward and the diaphragm^49,50^.

Expanding the scope of this study could lead to fruitful avenues for further research. Investigating the utility of advanced deep learning architectures, such as convolutional neural networks and recurrent neural networks with attention mechanisms, could potentially enhance the accuracy of predictions. Employing larger and more diverse datasets could strengthen the generalizability of the findings, ultimately benefiting patients with functional sexual disorders. Combining machine learning and deep learning techniques in ensemble learning configurations, such as stacking and boosting, might further refine the predictive capabilities of the models. Exploration of explainable AI tools could foster a better understanding of the underlying mechanisms driving the predictions, thereby increasing user trust and adoption. Addressing these topics could pave the way toward more informed decisions in managing functional sexual disorders, promoting better patient outcomes and satisfaction.

## Limitations

Predicting changes in core muscles during FSD using machine and deep learning techniques has the potential to improve diagnosis and treatment for women suffering from this condition. However, while the use of these techniques shows promise, several limitations need to be considered. These limitations include issues such as small sample sizes, limited accuracy, limited availability of data, and ethical considerations. Understanding these limitations is crucial for developing accurate and effective models for predicting changes in core muscles during FSD and ensuring that these models are used ethically and responsibly. In this paper, we will explore the limitations of predicting changes in core muscles during FSD using machine and deep learning techniques and discuss the implications of these limitations for future research and clinical practice.

- Small sample size: the study had a relatively small sample size, which may limit the generalizability of the findings.
- Lack of diversity: the study included only a limited number of participants, which may not accurately represent the diversity of the population.
- Lack of consideration for psychological factors: the study focused solely on changes in core muscles during FSD and did not consider other potential factors such as psychological factors that may contribute to the condition.
- Limited scope: the study only focused on predicting changes in core muscles during FSD on and did not explore other potential applications of machine and deep learning in the field of sexual health.
- Limited accuracy: while the study found that machine and deep learning algorithms can be effective in predicting changes in core muscles during FSD, the accuracy of the models may still be limited.
- Limited availability of data: the availability of data on core muscle activity during FSD may be limited, which could affect the accuracy of the models.
- Limited access to technology: access to technology and expertise in machine and deep learning may be limited, which could limit the widespread use of these techniques in clinical practice.
- Limited understanding of the underlying mechanisms: the underlying mechanisms of FSD are complex and not fully understood, which could limit the accuracy of models that rely on these mechanisms.
- Limited generalizability: the findings of the study may not be generalizable to other populations or contexts, which could limit the applicability of the models.

## Conclusions

Female sexual dysfunction (FSD) is a complex condition affecting many women, with symptoms like pain during intercourse, decreased libido, and difficulty achieving orgasm. Changes in core muscle activity, such as the pelvic floor muscles and diaphragm muscles, may contribute to FSD, but predicting these changes accurately is challenging. This study explored machine and deep learning techniques to predict changes in core muscles during FSD. Four deep learning (MLP, LSTM, CNN, RNN) and four machine learning algorithms (ElasticNetCV, random forest regressor, SVR, and Bagging regressor) were evaluated based on their performance metrics. The results suggest both types of algorithms can effectively predict changes in core muscles during FSD, with machine learning being faster and deep learning being more accurate. Future research may explore additional algorithms and techniques to enhance accuracy, recognizing predictive factors and identifying subgroups of women who are more likely to develop comorbid conditions will probably make it easier to put preventative measures in place and improve management, which will help lower the socioeconomic costs related to these common medical issues.

## Data Availability

The dataset and code used in this study is public and all test data are available at this portal (Click Here); Link 1: https://shorturl.at/qswKO; Link 2: https://rb.gy/y0vqw.
